# Building resilient partnerships: How businesses and nonprofits create the capacity for responsiveness

**DOI:** 10.3389/frhs.2023.1155941

**Published:** 2023-05-15

**Authors:** Lauren A. Taylor, Emma-Louise Aveling, Jane Roberts, Nazmim Bhuiya, Amy Edmondson, Sara Singer

**Affiliations:** ^1^ Department of Population Health, NYU Grossman School of Medicine, New York, NY, United States; ^2^ Department of Health Policy and Management, Harvard TH Chan School of Public Health, Boston, MA, United States; ^3^ Survey and Qualitative Methods Core, Division of Population Sciences, Dana–Farber Cancer Institute, Boston, MA, United States; ^4^ MassHealth, Executive Office of Health & Human Services, Boston, MA, United States; ^5^ Harvard Business School, Boston, MA, United States; ^6^ Department of Medicine, Stanford University School of Medicine, Stanford, CA, United States

**Keywords:** business, nonprofit, strategic partnership, community health, management, health equity, sustainability

## Abstract

Increasingly, businesses are eager to partner with nonprofit organizations to benefit their communities. In spite of good intentions, differences between nonprofit and business organizations can limit the ability of potential partnerships to respond to a changing economic and public health landscape. Using a retrospective, multiple-case study, we sought to investigate the managerial behaviors that enabled businesses and nonprofits to be themselves *together* in sustainable partnerships. We recruited four nonprofit-business partnerships in the Boston area to serve as cases for our study. Each was designed to address social determinants of health. We thematically analyzed qualitative data from 113 semi-structured interviews, 9 focus groups and 29.5 h of direct observations to identify organizational capacities that build resilient partnerships. Although it is common to emphasize the similarities between partners, we found that it was the acknowledgement of difference that set partnerships up for success. This acknowledgement introduced substantial uncertainty that made managers uncomfortable. Organizations that built the internal capacity to be responsive to, but not control, one another were able to derive value from their unique assets.

## Introduction

Amidst intensifying racial, economic, environmental and health crises, businesses are facing increasing pressure to demonstrate to various stakeholders that they are taking their social responsibility seriously (1). One way to do this is to develop relationships with nonprofit organizations whose full-time job it is to undertake community improvement initiatives such as improving access to nutritious food, educational opportunities for Black and Brown youth and air quality in cities. The quality and nature of these relationships can vary widely (2–7). In some cases, the relationship is largely transactional and involves only the occasional transfer of funds to a portfolio of grantees. In other cases, partnerships can last many years and involve many more touchpoints. Business-nonprofit relationships are a topic of enduring academic interest. To date, scholars have established that these relationships are challenged by the competing institutional logics in nonprofit vs. business sectors and power imbalances derived from financial asymmetries (8–12). Managers are willing to endure these challenges on the basis that the relationship offers each partner access to novel resources (e.g., funds but also potentially networks, expertise, social capital etc) within the other (13, 14). Yet resource dependencies may also intensify the adverse impacts of business and nonprofits’ divergent logics (8).

The existing literature points to two approaches to effectively managing the competing logics inherent in business-nonprofit relationships, which can appear contradictory. One approach, emphasized in the practitioner literature but found in some scholarly writing, asks business and nonprofit partners to engage in advanced planning to ensure that goals and work plans are shared by all involved. In one instance, authors *define* a meaningful partnership as commitment to a common goal, including joint provision of resources and sharing of risks “that was directed from the outset (15).” One of James Austin's early, seminal works on the topic lends credibility to this approach, suggesting that “The more specifically one can articulate expected benefits at the outset, the greater guidance the partnership will have (16).” This approach often imports an assumption that partnerships can be understood as having a life-cycle, wherein they progressively deepen over time until dissolution at the discretion of the management team. Moreover, it implies that the business and the nonprofit should be able to employ strategic management techniques to be the masters of their own fate (17).

A second approach for managing the inherent complexity of business-nonprofit relationships focuses on the need for continual learning and a more emergent approach to planning (18, 19). This approach emphasizes *differences* between partners as the organizing principle, wherein the value of partnership lies in its ability to exploit and capitalize on these differences. As such, the inherent tensions of partnership work are an inescapable pre-requisite, and respecting, rather than erasing, difference should be a central managerial objective (20) towards developing a resilient partnership. This more exploratory approach to managing business-nonprofit partnerships allows a role for environmental uncertainty and assumes less about the way in which the relationship may become more, or less, integrated over time. In this sense, the business-nonprofit partnership literature is following recent work on the need for greater flexibility in order to ensure the success of for-profit joint ventures (21).

Our work draws from this second approach to managing the inescapable tensions in business-nonprofit partnerships for the purposes of health improvement. Each of our case studies began with an acknowledgement that businesses and nonprofits often offered radically different working environments. Before partners could leverage those differences, they needed first to be acknowledged and explored (20). Importantly, we found it counterproductive, if not impossible, for business and nonprofit partners to try and erase these differences. Previous studies have identified general inter-organizational processes for managing partnerships that are premised on difference, such as building trust, enabling communication and facilitating mutual understanding. Some have advocated partnering entities to develop capacities for learning or partnering across organizational boundaries (18). Considerably fewer have specified the intra-organizational capacity that businesses and nonprofits must develop to manage successful inter-organizational partnerships.

Although the public discourse on health services frequently references the value of partnership—as well as related terms such as collaboration and coalition—indiscriminate use of the term “partnership” to describe a broad swath of collaborative engagements has muddied the water when it comes to identifying the challenges and solutions to establishing and sustaining specific forms of engagement. Indeed the word has been used in reference to everything from contractual or vendor-style relationships to long-term, deeply collaborative relationships. Studies of public-private partnerships primarily include governments and businesses, overlooking the nonprofit sector, lending further opacity to any rhetorical shorthand. We focus our analysis on what we call “strategic partnerships” between businesses and nonprofits rather than the less intensive, but more common grantor-grantee relationships. We define strategic partnerships as inter-organizational collaborations which are deliberately undertaken to advance the positon of participating organizations. Doing so is appropriate for the way in which many businesses are re-conceptualizing their philanthropic or corporate social responsibility (CSR) engagement away from a portfolio approach and towards fewer and deeper alliances. High-profile strategic partnerships include Google's work with the Trevor Project, a confidential crisis text line for LGBTQ youth (22), and Timberland's relationship with educational nonprofit City Year (23). These “fewer, deeper” partnerships often include contributions from the business that extend beyond financial commitments, including board memberships, volunteer opportunities for employees, matching contribution programs, and co-branding opportunities.

While prior research has focused on the initiation and early stages of the partnership (24), reviews of cross-sector collaborations emphasize the need for longitudinal case studies rather than point-in-time research to illuminate the dynamic nature of partnerships and lend greater insight into what makes the arrangement sustainable over time (5, 25, 26). We aim to partly fill this gap by summarizing the findings of our retrospective investigation of four strategic partnerships between business and nonprofits, all of which had existed for 6–10 years at the time of study. The substantial duration of collaboration, in the face of inevitable environmental and organization-specific changes, is what made the partnerships “resilient” in our view. While this study is not prospectively longitudinal, our cases were selected and data collection instruments were designed to harvest insights from businesses and nonprofits that had been in relationship with one another for several years.

We set out to investigate what makes businesses and nonprofits successful in building and sustaining strategic partnerships with one another. To do so, it was critical to first confront that the cultural and cognitive distance between business and nonprofit organizations. This distance presents challenges above and beyond those typically found in business-to-business joint ventures or strategic partnerships (27–30). By dint of their differences from one another, even skilled and well-intentioned managers found this work difficult. We outline the challenges associated with business-nonprofit partnerships, which largely confirm previous findings, in Part 1.

In Part 2, we outline lessons for how businesses and nonprofits can develop the capacity to be an effective strategic partner. These insights run counter to much of common managerial practice. We found that managers' willingness to accept an open-ended future for their relationship with nonprofits was key to their success over the long term but also introduced an uncomfortable element of uncertainty. The very same disruptions that spurred these partnerships may challenge both partners' ability to meet their equity commitments. The COVID-19 pandemic has presented economic challenges to business and nonprofit organizations alike (31) In order to build resilience in the midst of this uncertainty, businesses and nonprofits needed to develop the capacity to be responsive to their partners. We surmise that standard accounts of business-nonprofit relationships have overlooked, or at least downplayed, the need for *both* partners to develop new capacities in part because analyses undertaken through the lens of resource dependence so often magnify the financial dependence, and therefore willingness to change, of the nonprofit partner (18). In contrast, the business is imagined to be a resource*ful* and therefore more static partner. Our fieldwork indicated that this is an oversight and the business' intra-firm development was just as important as the nonprofits' intra-firm efforts or the inter-organizational practices that have been described at some length by others. Based on our fieldwork, we provide several examples of how successful strategic partnerships built this “capacity for responsiveness” internally.

## Materials and methods

In this paper we summarize findings based on analysis of four case studies examining the role of cross-sector collaboration as a means to promoting heath equity in the city of Boston (Table 1). We analyzed qualitative data from 113 semi-structured interviews with business, nonprofit, and public sector leaders and employees from 42 organizations involved in long-term collaborative initiatives. Additionally, we conducted 10 focus groups. 9 of those focus groups were with Boston public school teens and young adults (*n* = 40) who used, or were impacted by, the services or activities offered by case study initiatives. 1 focus group was with a group of employees of the retail nonprofit operation in Case 2. We did no focus groups in Case 4 because the population whose health was targeted for improvement were young (elementary school) children. Finally, we conducted 29.5 h of direct observations of initiative activities (e.g., service delivery activities, stakeholder meetings), which provided additional perspective on the nature and mechanisms of collaboration.

**Table 1:** Data collection by case.

**Case 1—The Arm's Length Model** Financial Services Business and Nonprofit Partners *Data collected Winter 2018–Winter 2019*			
* *	Organization	Role (s)	*n*
*Interviews*			
** **	Focal for profit business	Senior leaders, managers (CSR, Marketing depts.)	11
** **	Focal nonprofit partners	Senior leaders, frontline staff	24
*Focus groups*			
* *	Nonprofit partners	Youth/young adult initiative participants	6 groups
*Observation*			18.5 h
**Case 2—The Operational Partnership Model** Retail Nonprofit (Generated revenue through sales; registered as a 501c3) *Data collected Spring 2018–Winter 2019*			
* *	Organization	Role (s)	*n*
*Interviews*			
** **	Focal nonprofit	Senior team, middle management	8
** **	** **	Board members	3
** **	For-profit partners	Directors/senior leaders	2
** **	Nonprofit partners	Senior leaders	4
*Focus Groups*			
* *	Focal nonprofit	Employees	1 group
*Observation*			5 h
**Case 3—The Incubator Model** Industrials Business and Education Nonprofit *Data collected Spring 2018–Winter 2019*			
** **	Organization	Role (s)	*n*
*Interviews*			
	Focal for profit business	Senior leaders, staff	6
	Focal nonprofit organization	Senior leaders, middle management, frontline staff, board members	12
	Public sector organizations	Senior leaders, staff	7
*Focus groups*			
	Nonprofit/public sector	Youth/young adult program participants	3 groups
*Observations*			6.5 h
**Case 4—The Adoption Model** International Apparel Business and Health Nonprofit *Data Collected Spring 2019–Winter 2019*			
** **	Organization	Role (s)	*n*
*Interviews*			
	Focal Nonprofit	Senior leaders, managers, staff	9
	Focal For-profit business partner	Senior leaders, staff, board members	6
	Public sector organizations	Senior leaders, staff	9
	Nonprofit partners	Senior leader, manager, board member	4
	For profit funding partners	Senior leaders, staff	4
*Observation*			5 h

We identified candidate cases *via* extensive web-searches of organizations and nonprofits engaged in strategic partnerships targeting health and well-being, triangulated with information from discussions with locally knowledgeable members of Harvard University's business and public health communities, the local business and philanthropic community, city officials, and the study's advisory council, which was comprised of leaders of business, non-profit organizations and consortia from across the country.

Our research team selected case study candidates based on a series of criteria which were refined over the course of our case identification process in an effort to balance the focus and breadth of our study. We ultimately decided that in order to be considered, partnerships needed to demonstrate the following: (1) *locale*: the partnership must be between Boston-based organizations and focus on improving the conditions of the Boston community, (2) *composition*: the partnership must have involved at least one business and one or more nonprofit organizations (and may include government), (3) *minimum level of engagement*: the partnership work must have entailed more than financial transfers (eg. not solely a philanthropic funding relationship) (4) *duration*: the partnership must have been ongoing for several years and (5) *novelty*: the partnership must not have been previously studied by an academic research team. These criteria were chosen in order to keep certain elements of the research context consistent. We also sought to achieve diversity amongst the cases in two, major respects: (1) *level of integration*: the nature of the relationship between the business and nonprofit partners at the point of data collection and (2) *social determinant of health focus*: the social determinant of health focus of the collaboration (e.g., nutrition, physical activity etc).

The research team used its judgement to determine how many and which inclusion criteria would narrow the scope of our inquiry sufficiently to develop new insights about business-nonprofit partnerships. The axes of difference were chosen to reduce the risk of observing and interpreting insights about a specific level of integration or social domain as a generalizable finding about the more general category of business-nonprofit partnerships. Using our judgement to determine the appropriate selection parameter introduces a source of bias to the design but is widely accepted in qualitative research that is intended to generate insight rather than test relational hypotheses. These insights can be subsequently tested in quantitative analyses with sampling strategies better suited for causal inference.

**Figure 1:**
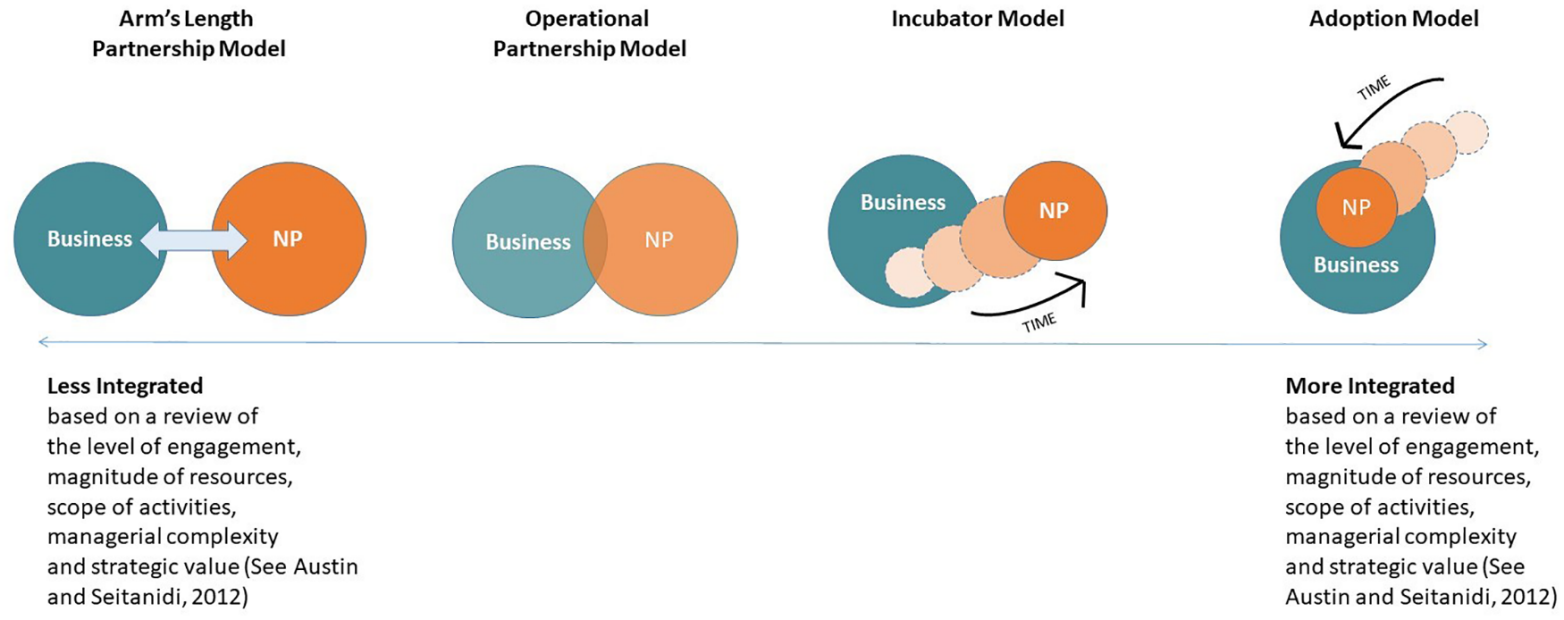
Continuum of business-nonprofit integration.

The four resulting cases differed in terms of their level of organizational integration and can be situated on a continuum (Figure 1). We use the terms “more” and “less” integrated throughout in reference to the intensity of collaboration between the partners. In doing so, we draw on James Austin's work outlining a litany of dimensions on which integration varies, including (but not limited to) the level of engagement, magnitude of resources, scope of activities, managerial complexity and strategic value (32). We depart from Austin's previous work, however, in conceptualizing a continuum that carries no normative valence that one level of integration is better than another and no expectation that a partnership will progress through orderly stages. As we later describe, our data included partnerships that moved from right to left and from left to right and both were viewed as successful by the relevant stakeholders.

Our first case, which we refer to as the “The Arm’s Length Partnership Model” involved a national financial services business and a handful of longstanding nonprofit grantee partners. The business' relationships with the grantees began as primarily financial but grew in such a way that business employees volunteered their time to “consult” with nonprofits and made themselves available to serve on nonprofit boards. Our second case, termed “The Operational Partnership Model” involved a retail nonprofit (registered as a 501c3) which fulfilled its mission, and generated its revenue, by selling goods donated from local businesses. We refer to this as an operational partnership because the nonprofits mission was dependent on the regular weekly or monthly participation of local businesses, as well as significant funding from a corporate foundation. We refer to our third case as “The Incubator Model”, as it was comprised of a national industrials business, which had established a nonprofit initiative within its own organization and ultimately span it out. The two organizations maintained a close working relationship, with the business still providing some financial and operational support and business members involved as volunteers and board members. Our fourth case, “The Adoption Model”, involved a nonprofit initiative that became embedded within a for-profit international apparel company, which also acted as its landlord and main funding partner. To protect the identity of the partnerships we provide limited details on the organizations involved.

For clarity, we have labeled our cases based the nature of business and nonprofit integration. Doing so suggests a dyadic focal relationship. In reality, we observed the focal organizations as embedded in often complex networks of nonprofit, for profit and municipal organizations. Diagrams illustrating the complexity of these networks for each case are available in the Appendix.

A note on the public sector's involvement across the project is warranted. The public sector, primarily in the form of city government, played a role in shaping each of these partnerships. In Cases 3 and 4, that role was operational in the sense that a public institution served as a site of partnership activity or a gatekeeper to key constituencies (e.g., school-aged children), hence the public sectors' inclusion in the interview set. In the other two cases, the public sector was not an active participant. All business' involved in our fieldwork were cognizant of the public sector, and particularly people in government with regulatory power, as an important audience for their partnership work. That said, key informants understood themselves to be primarily in partnerships between nonprofit and for-profit organizations, which is why we chose to reflect that language and emphasis throughout.

Table 1 provides further detail on the data collection for each case. After obtaining agreement from each of the collaborating organizations involved in the four cases, we identified individual participants and opportunities for observation in consultation with the host organizations. The interview sample included representatives from the business and nonprofit organizations involved in each partnership, and from purposively selected organizations with more peripheral involvement. Those organizations with more peripheral involvement included other businesses or nonprofits involved in sponsoring or funding initiatives as well as public agencies. Interviews were conducted in-person or by Zoom at the key informants' convenience and lasted 30–60 min. Interview guides focused on eliciting key informants' perspectives on the nature of their involvement with the partnership, origin and evolution stories of the partnership, motivations for partnering, and challenges faced and benefits gained from the relationship. Focus groups, which we conducted to elicit the perspective of clients, service users and beneficiaries, focused less on operational tactics and more on their perceptions of the business and nonprofits in question and the effects of the partnership. Each focus group was facilitated by two members of the independent research team, which included experienced qualitative researchers, and a university community liaison director who was also a former youth worker. All participants consented to participate in the focus groups, and parental/guardian permission was sought where appropriate.

We analyzed the data within and across cases using principles of reflexive thematic analysis (33–35), which allowed us to identify and refine common and deviating themes through an iterative process of constant comparison. This approach emphasizes the importance of researcher's subjectivity as an analytic resource, rather than assuming that researchers' subjectivity is an obstacle to be avoided. We followed the process described by Braun and Clarke, including (1) data familiarization, (2) systematic data coding, (3) generating initial themes from coded data, (4) developing and reviewing themes, (5) refining, defining and naming themes and (6) writing a report (34). We coded for both semantic (overt) and latent (implicit) evidence in our data, with an eye towards key challenges faced by the strategic partnerships and effective strategies to overcome them. Throughout this process, our team used their judgement to elevate certain patterns and ideas, while relegating others. The role for researcher judgment allowed previously published frameworks and theories with which the research team was familiar to influence the analytic process. As a result, we consider our approach to be abductive, rather than purely inductive or deductive. In presenting the data herein, we use illustrative quotes which have been anonymized to preserve confidentiality.

We chose a case study research design in order to explore the in-depth dynamics of longstanding business-nonprofit partnerships. The tradeoff we made in selecting this research method is that some aspects our findings are not necessarily generalizable to cross-sector collaborations in other times, places and types of partnerships. For instance, drawing our cases from a single metropolitan area (Boston) may have influenced the behavior of businesses or non-profits in ways we could not detect without a comparator.

## Results

We first illustrate the reasons strategic partnerships between businesses and nonprofits can be difficult to develop and manage effectively. We then suggest several ways for managers to build capacity for responding to these challenges. Our case studies suggested that building sustainable business-nonprofit partnerships required each organization to cultivate the capacity to be responsive to their partner and their environments. Cultivating the capacity for responsiveness enabled partnerships to enact the learning over time, as previous studies have suggested is prudent (2, 16). Most critically, an approach that assumed uncertainty and adopted a strategy premised on responsiveness facilitated the capture of value from partner organizations' differences. While operating so flexibly may sound like a fly-by-the-seat-of-your-pants strategy, it is not: it requires considerable investment of resources and tactical decision-making, as we describe below.

### Part 1: Challenges to sustaining partnerships between nonprofits and business

Partnerships between business and nonprofit organizations entailed collaboration across divergent norms, practices, and ways of engaging with other organizations, which reflected the different sectors and relational contexts in which these organizations operate. The need to bridge diverse logics was central to understanding the distinctive challenges of building resilient, cross-sector partnerships (2, 10). We identified four central challenges faced by organizations engaging in cross-sector partnerships, which reflect the inherent unpredictability and gaps in mutual understanding that characterize efforts to build lasting, strategic partnerships. Identifying the nature of these challenges is essential to understanding what is required to be successful in partnerships (Table 2).

(1) How to manage shared work while respecting differences in structure, culture, and values across organizations

**Table 2:** Managerial challenges presented by business-nonprofit strategic partnerships.

Common assumption	Observed reality	Resulting managerial challenge
Partnerships are premised on shared goals and purpose across organizations (6, 15, 36)	Partnerships created value based on integrating differences between partnering organizations	Managing shared work while respecting structural, cultural, and values differences across organizations
Roles and expectations should be specified at the outset (6, 16, 37)	Lack of clarity at the outset was unavoidable because both parties lack critical information about the other	Establishing and conduct joint work without clarity on roles or expectations
Partnerships are dyadic, meaning between two entities (e.g., one business and one nonprofit) agreeing to work with one another (25, 38)	Both businesses and nonprofits were embedded in broader networks of organizations that exert influence on the partnership	Managing relationships with organizations embedded in public, for profit, and nonprofit networks
Progression of successful partnerships is to naturally deepen and become more integrated over time (32, 38, 39)	Deepening and becoming more integrated over time was not the only productive path forward	Managing a partnership with an open-ended trajectory

It is common to assume that nonprofits and businesses form strategic partnerships out of a sense of shared goals and purpose. To do so emphasizes the similarities or likenesses between the organizations as the basis for collaboration (15, 36). While partnerships entailed collaborating to achieve a joint operational goal (e.g., delivering a fitness program in schools, providing access to affordable fresh produce), our case studies suggested that successful management of these relationships relied on both partners also leveraging the underlying differences between them. These differences created the value proposition for partnering.

Together, successful partners strove to achieve goals that would have been impossible or at least difficult to achieve without one another. Nonprofits were keen to work with businesses on account of their assets, which included funding, relationships with local elites and access to in-kind resources (e.g., volunteers, operational support) and forms of expertise that nonprofits lacked (such as marketing or digital expertise) (40, 41). Businesses were attracted to working with nonprofits based on their strong relationships and legitimacy with local communities, and the relational expertise and technical expertise needed to deliver programs and maintain stakeholder engagement. One business manager described the know-how they gained through partnering as follows:

*“We also have [a relationship with Nonprofit] who helps provide the youth development perspective that we frankly, at [Business], we don’t have. I mean we’re a financial services company. We don’t know.”* (Business Manager)

That strategic partnerships are premised on difference created a managerial challenge: specifically, how to successfully navigate those differences. The business sector is generally characterized by a market logic that emphasizes competition and financial returns, while the nonprofit sector may be defined by a logic that emphasizes community responsiveness, health equity, and long-term time horizons. The varied levels of integration amongst the cases allowed for varying degree of separation between the organizations and their respective worldviews or institutional logics (8, 10, 42)*.* As the literature anticipated, less integrated relationships were less complex to manage but all partnerships faced some degree of logic conflict (40, 41, 43).

Managers on both the business and nonprofit sides of partnerships frequently drew stark comparisons between their organizations and ways of working. A nonprofit sector manager's made this emblematic comment:

*One of the things is they are a big corporation and we are a smaller grassroots program. We're so far apart sometimes […] I think people just don't understand how things operate between two worlds all the time.* (Nonprofit Manager)

These logics inform the principles, expectations, and norms that in turn shaped the behaviors, priorities, and understandings of people working in different sectors.

The divergent logics sometimes manifested as tension within the partnership. In one case, tension bubbled up over the importance of branding consistency and justifiable uses of money within the partnership. A senior manager of a youth serving nonprofit described the challenges that a logo change presented to their organization, contrasting the experience with what they understood of a business' experience of the same kind of change:

*For a youth serving nonprofit, consistency is so key to having a brand recognized by young people … So [when you get a new logo] it’s like, Now you have to change the uniforms, you have to change your t-shirts, your materials. For a private sector, it’s like, “Oh, yeah, we can do the design and then we can just order them.” … In the nonprofit sector, those are dollars to undo, that we could actually be dedicating to direct service.* (Nonprofit Senior Manager)

This culture clash had relational consequences*,* as misunderstandings and misinterpretation of partner behavior undermined trust. For many businesses entering into collaborations with nonprofit partners, a trust deficit may be present from the very start and therefore exacerbate misunderstandings due to cultural differences. One interviewee reflected on how nonprofit sector colleagues negatively perceived business:

*I have definitely become much more of a believer of that business community can or should be more a part of addressing issues. I don’t know if anyone ever said this to me explicitly, but being in education and community for so long, business was [seen as] just … bad. You didn’t even try to engage businesses in the community. Most people's orientation is that “I don’t want anything to do with business”.* (Nonprofit Employee)

(2) How to conduct work without clarity on roles or expectations

Good managerial practice often requires specifying roles and end goals at the outset of a project. A hallmark saying of strategists is “start with the end in mind (44).” The implication is that planning can proceed backwards from a clear picture of a desired outcome. Business managers often approach their relationships to nonprofits in this way. This thinking has migrated into previous writing about strategic, cross-sector collaborations. Don Barr, for instance, defined partnership as a commitment to a common goal, including joint provision of resources and sharing of risks, “that was directed from the outset.” He and others presumed that clarity will mitigate the potential for conflict (15).

What we observed in our cases, however, was that both the final products as well as various players' roles were often impossible to gauge accurately at the start. The relationship between organizations often took root prior to a precise understanding of what the work might entail, often because people in positions of authority had met and developed a relationship or engaged in low-intensity forms of collaboration before committing to a more substantial organizational partnership. As a result, the process of identifying areas of alignment and partners' strengths and capacities unfolded gradually after kickoff events and public announcements.

*At the beginning it was, it was still partnership [but] it became more substantial because we really [came to share] the development of the program. And this is something that [Business] does with a number of other organizations [..] So, their attitude is a bit different than just having a kind of, “Well, here is the partnership. Here is how it's going to work. You’re going to do X. We’re going to do Y and that's the end of it.”* (Nonprofit Employee)

Because these long-term relationships were constantly evolving, managers were challenged to clarify roles and end goals sufficiently to enable work, but not so dramatically as to stymie change. In one case, nonprofit leadership shared with the research team that they had initially agreed that the nonprofit was the business's signature CSR commitment but were now trying to determine what their organization's role would be in terms of encouraging the business’ non-CSR employees to consider the social impacts of their products. This was a delicate matter for the nonprofit, which was eager to push the business towards more pro-social action but conscious to avoid doing so in a way that would inadvertently sour the business leadership's commitment to the cause.

Although some unpredictability is inherent in all partnerships, even in those between businesses, the nonprofit context offered special challenges. Nonprofits strived to be community-responsive, meaning that their programmatic foci and potentially even their missions, are subject to change.

*My sole focus is about what else can we give to our young people to make them successful. And we're going to do whatever we have to make sure that that is happening… What that looks like [in practice], it's going to take different shape and form. Their needs are changing on a regular basis.* (Nonprofit Leader)

The challenge for business managers was to conduct work without precise role and timeline definitions.

(3) How to manage relationships with organizations embedded in public, for profit and nonprofit networks

Particularly in the practitioner literature, partnerships are assumed to exist between two entities. A Google search for the term “partnership” conjures hundreds of pictures of two people holding hands, shaking hands, and connecting puzzle pieces. All convey an image of partnerships are dyadic. Business managers who sign Memorandums of Understanding (MOU) or Business Affiliate Agreements (BAA) with nonprofits often make this same image in mind.

The assumption that a business-nonprofit relationship is dyadic can set businesses, in particular, up for frustration. Businesses in our study that signed an MOU or BAA with a single nonprofit ultimately found themselves in relationships with considerably more groups by virtue of the nonprofit organizations' embeddedness in broader networks. One nonprofit manager descried the web of accountability and therefore network ties their organization faced:

*I think we’re certainly accountable to our service-users, their families, and the [municipal government department] as well as our funders …. From my perspective, if you’re engaging with your community and you’re putting a potential solution out there and you’re taking funds in support of that solution or that mission, you’re accountable to quite a cross-section of people.* (Nonprofit Senior Leader)

Similarly, one nonprofit with three sites explained how it had multiple funding partners:

*Well, [our work is] funded differently in each of its three sites, but in [Location 1] it’s funded by a combination mostly of city money, a little bit of state money. [Location 2] is funded through some state money, a tiny bit of city money, and mostly private money, and [Location 3] is sort of similar to [Location 2] in that it doesn’t have a whole lot of governmental money and it's mostly private money. We also do have relationships with [Business], maybe some other corporations, and we make some money through our consultancy.* (Nonprofit Senior Manager)

These ties, which might include other major funders or key implementing partners, stood to unexpectedly influence nonprofit partners in ways that impinged on their relationship with the business partner. A business in our study, for instance, was engaged with a nonprofit that was also working with the local public school system. In the eyes of the nonprofit, it was the public sector partner that was most essential to the nonprofits' existence:

*I think the most key relationships for us in order to continue to exist are the school districts. I mean, the districts themselves, the headmasters of each of the individual schools that we’re at, the coaches, the teachers at those schools, those are the people that are our stakeholders and that we have to continue to engage and demonstrate our value to.* (Nonprofit Manager*)*

This relationship to the public school system exposed the nonprofit to a series of political and bureaucratic decisions made by people outside of its organization. At the outset, the business partner did not understand the extent of the *other* partner's influence on the nonprofit partner's priorities, needs, and practices. Further, as the nonprofit scaled up and expanded into new locations, still additional influences were added over the course of the partnership. New partners introduced additional uncertainty and potential for misunderstandings for the original business partner, as the introduction of additional collaborators risked compromising the autonomy of the nonprofit.

In one case, the embedded nature of the nonprofit work actually constrained how the business-nonprofit partnership was able to scale up. Both parties were interested in seeing the nonprofit's work reaching additional people in new communities. In furtherance of this goal and its own standing as a prominent CSR player, the business would tout the benefits of the nonprofit's programming to local governments when it moved into a new community. In the minds of the business' leadership, doing so was at least partly a favor to the nonprofit insomuch as it advanced the nonprofit's reputation. However, the nonprofit was reliant on the participation of other stakeholders, beyond the business' purview, in order to successfully establish their program in new communities. As a result, the nonprofit and business agreed that scaling their two operations into new geographies in tandem would be difficult. Instead, the nonprofit would have to trail the business' expansion and consider each community on a case-by-case basis. Coming to terms with this approach required lengthy and careful discussion between partners, including the development of clearer criteria for scaling up to avoid damage to the nonprofit's relationships with the business as well as other key stakeholders in its network.

The uncertainty that stemmed from this sort of embeddedness within wider relational networks is inherent to collaborations with nonprofits. The uncertainty may be particularly challenging for a business when the business itself has no direct relationships with, or limited understanding of, the expectations, practices, and priorities of those other influential players.

(4) How to manage a partnership with an open-ended trajectory

A substantial thread of the partnership literature assumes that partnerships will naturally deepen and become progressively more integrated (15, 38). Moreover, the implicit assumption is that increasing closeness or integration is desirable—a reflection of a successful partnership, while greater separation over time indicates regression or failure (16, 45).

We found, however, that moving toward deeper integration is not the only “successful” trajectory for relationships between business and nonprofit partners. Instead, our study found evidence that a strategic partnership may grow *less* integrated over time but nevertheless be considered successful by partners.

The “Incubation Model” case provides an example. The nonprofit began as the CSR initiative of the industrials' business, but now operates as an independent 501c3 focused on physical fitness. After several years operating as an “in-house” initiative that staff volunteered time and money to, the initiative grew in scope and became increasingly organizationally independent from the business. It was eventually spun out as a standalone entity to allow the nonprofit to attract additional resources from philanthropic funders. Today, the two organizations are still closely engaged with one another. The business also continues to be one of the nonprofit's major funders, remains involved in its strategic development, and plays an important role on its board of directors. Both the business and the nonprofit viewed this development as a success. New research indicated that this kind of “spinout” is becoming an increasingly common pathway for ending business-nonprofit relationships and a potentially attractive alternative to exit *via* “dissolution (46).”

Even so, out data indicated that the spinout evolution created challenges for managing the relationship, as the business had to figure out how to “let go” of the nonprofit that had been born within its four walls. Business managers remarked:

*I think it's having that balance of still having a connection and still being visible, but from a structural and resource standpoint [allowing the nonprofit to] stand more on its own.* (Business Senior Leader)

*We're trying to let this [new] board come in, get involved. I think we would love to play a continuing role, but at a smaller level so that it can actually grow and achieve what it can achieve.* (Business Senior Leader)

Successful collaborative trajectories can take many paths and business cannot know at the beginning which path will be most advantageous or which values or aspects of shared vision may shift over time (21, 29, 47)*.* The potential for successful partnerships to travel in more than one direction added to uncertainty, as practitioners lack a reference trajectory for how success should be defined in advance.

### Part 2: Building capacity for responsiveness

Although managers cannot eliminate the differences, uncertainties, or unpredictability described above, we identified four ways in which organizations in our caes studies reformed (or failed to reform) themselves internally so as to position themselves for success. Each reform involved building an organization's capacity to respond to partners and their environments—this is resilience. Note that the goal was to create an organizational environment where managers could be responsive to a partner whose differences were *respected*—rather than controlling partnera whose differences were *resented*. We therefore refer to the package of four approaches as the “capacity to be responsive (Table 3).”

**Table 3:** Managerial challenges and suggested actions

Managerial challenges borne of uncertainty	Suggested actions to build a capacity for responsiveness
• How to manage shared work while respecting structural, cultural, and values differences across organizations	• Develop a set of minimum viable conditions for the partnership—otherwise, be willing to tolerate ambiguity and uncertainty
• How to conduct work without clarity on roles or expectations	• Create structures for mutual dialogue up and down, within and between organizations
• How to manage relationships with organizations embedded in public, for profit, and nonprofit networks	• Recruit and develop people with experience in both business and nonprofit domains to navigate and leverage organizations’ different strengths
• How to manage a partnership with an open-ended trajectory	• Mobilize non-financial commitments in support of the partnership

In our usage, to be responsive means to identify and accommodate differences and uncertainties intrinsic to strategic partnerships. Our general finding that the management of uncertainty requires organizations to commit substantial resources to “governance” accords with previous transaction-costs literature on public-private partnerships by Rangan, Samii and van Wasserhove (48). We describe the specific steps in more detail below to convey how partners can build internal resilience to nurture sustainable relationships.

(1) Develop a set of minimum viable conditions for the partnership—otherwise, be willing tolerate ambiguity and uncertainty

In contrast to many managers' instincts, our findings demonstrate that collaborations can profitably begin with considerable ambiguity and evolve over time. Roles and contributions in longer-term partnerships need space to grow and change in order for the partnership to remain relevant for partners and effective in problem solving. While many managers recognize the conceptual need for such openness, arranging workflows and business processes to support it can feel slow, if not circular. In our study, partners that were able to maintain a productively open stance were those that had developed and could communicate a set of “minimum viable” conditions for the relationship (16, 49). Minimum viable conditions refer to the “must haves” that each party requires for the partnership to be acceptable. Identifying these early allows both partners to avoid wasting time and resources in a relationship that will ultimately derail.

*I think that unfortunately, sometimes, as a nonprofit, you are faced in that position, where like you really need this grant. But then there's one kind of piece that [the business] wants to see added to a project plan. Then, all of a sudden, you have this like mission creep, [..] And I think, you know, to [Business'] credit, I think that they’re very clear about what their approach to CSR is. (*Nonprofit Manager*)*

The primary condition that needed to be recognized was the purpose for engagement—namely, what brings each partner to the table? Importantly, the purpose for business' participation in the partnership needed not be same purpose that nonprofits are pursuing. The respective rationales needed simply be identified and accepted by both parties. Though it could be tempting for business to withhold key information, such as an interest in reputation gains, from the conversation about purposes, we found that the clearest possible articulation of each party's rationales was critical to facilitating mutual understanding and anticipatory decision-making.

Other potential minimum viable conditions for the partnership flowed from the articulation of purpose. For instance, we observed a meeting in which a business partner set out its minimum requirements for engagement with its key nonprofit partners. Leadership from each of the nonprofits in the room were asked to sign a “partnership agreement” which included requirements for nonprofit partners to complete regular surveys and evaluations for longitudinal research being done on the nonprofit initiative. The business also established that consistent use of their logo in public facing materials was critical to sustaining support for the partnership within the business.

Apart from the articulation of minimum conditions, we found that successful partners in our study took a particularly developmental approach to managing their relationship, allowing the relationships to develop over time rather the specifying the form and extent of collaboration at the outset. In one case, the partnership proceeded gradually in expanding the scope of collaboration, roles and contributions over time, allowing mutual understanding to inform these changes. One senior business manager described their approach as follows:

*It's kind of figuring out what [nonprofit] need and where we can plug in because the last thing we wanna do is [..] try to jam something down their throat. That doesn’t help them. So I think that's what we are really good at, is trying to get a sense of what the nonprofits need and then above the grant, trying to fill in what those gaps are with employees and resources.* (Business Senior Manager)

In its relationship with one nonprofit, the business' support began with event sponsorship but morphed over time to be considerably more involved as a result of conversations with the nonprofit. Ultimately the nature of the partnership took a form that could not have been predicted. It also involved recognizing when collaborative projects were *not* working, and shifting gears appropriately. At one point the partners decided to end one aspect of their joint work when it proved a poor fit with service users' needs and constraints. This “ending” did not spell an end to the partnership but rather an impetus to find alternative ways to collaborate.

In pursuing pro-social work, it was inevitable that contexts and needs change and the nature of these changes cannot be known at the start. In one case, initially the partnership was squarely focused on acquiring equipment and space for youth fitness activities. Over time, in response to changing needs within the community being served, the partnership successfully shifted its focus to supporting the well-being of young people participating in the initiative:

*When we started there was a heavy investment getting the facilities and the uniforms and equipment to a standard which people thought was appropriate, and we don't do that anymore. [Nonprofit] doesn't do that anymore, because it has built up the infrastructure and now it's focusing on [other aspects of kids' health]. So, I think it's a lot deeper of a mission and intended outcome than when it started.* (Public Sector Partner)

This was facilitated by the willingness of the business partner to ask nonprofit partners and community members about their perception of where the need was greatest.

In both cases, strategic shifts were only possible because the managerial team's willingness to confront some degree of ambiguity about the future of the relationship with the nonprofit—including the possibility that it may end. In this way, resilient partnerships were those open change and communication between organizations.

(2) Develop two-way dialogue structures up and down, within and between organizations

Organizations in our study that developed multi-level, two-way dialogue within and between partners appeared more resilient amid the inherent uncertainties associated with strategic partnerships. Two-way dialogue refers to communication patterns that allow both parties to share and listen.

Communication *between* the business and nonprofit was understandably vital. One nonprofit leader summarized the importance as follows: “*There have to be, I think—very clear goals, clear communication, clear contact people. [As a partner, I want to know]– what is the structure of the flow of communication?”* (Nonprofit Manager) Nonprofit managers in particular described the importance of feeling that business partners sought and valued their input. Such dialogue *between* partner organizations was most effective when it occurred not only at one level (e.g., between frontline staff) but at multiple organizational levels. This intentional redundancy in communication limited the potential for misunderstandings and misinterpretations, which are otherwise common (50), and laid the foundation for the development of mutual understanding and responsiveness.

*It's really not a sponsor relationship, it's really not like we give you a bunch of money, and then you put our logo everywhere. Any time that the [Nonprofit] is doing something new, or we have a new set of goals to align with, we really come together and talk about that, about how we can both benefit from a partnership perspective.* (Business Manager)

A school principal, for instance, articulated the school staff's appreciation for the business partner's transparency and communication:

*They are extremely transparent, which allows us to make informed decisions about either continuing the relationship or redefining the relationship or dissolving the relationship, and I think that is important. The players may change, and they have, but the goal does not change. And that's how we survive.* (Public Sector Partner)

The development of two-way dialogue was dependent on the communication patterns and preferences of business leaders but was also a structural feature of the relationship. In our study, managers made structural commitments to facilitate two-way dialogue with the nonprofit by co-locating employees, establishing standing, formal meetings, and identifying “point people” within both organizations. Nonprofit employees shared with us that they valued the ability to pick up the phone and call a point person rather than waiting to raise something in a formally scheduled meeting.

Less intuitive but equally vital was communication *within* the participating organizations about the partnership. Communication up and down the business' internal organizational hierarchy was especially important in order to facilitate information transfer from front-line managers who were engaged in partnership activities to senior leaders making key strategic decisions and holding purse strings. Horizontal communication between partners was described as equally important:

*My point is in the organization there is the employee wellness, there is like the long term care wellness, there is life insurance wellness, there is the real estate aspect, corporate responsibility [all of whom are involved in this project]. So we just like once a month get together and kind of talk about what each group is doing.* (Business Manager)

Creating such communication flows stands in contrast to the more common business practice of isolating communication with and about nonprofits within CSR departments, but is consistent with previous literature highlighting the importance of mundane, relationship management work (3). Just as Nithin Nohria and his colleagues found in business to business partnerships, we found that managerial processes matter a great deal in determining the viability of business-nonprofit partnerships (51). We found that considerable frustration between partners could be avoided when communication channels within each of the partnering organizations was effective. One of our case study businesses was especially conscientious about the need to keep people within the company informed about opportunities to work directly with partner nonprofits. We spoke with an employee who was volunteering at a partner nonprofit's event about how they found out about that opportunity. They described the within-firm communication about the nonprofit partnerships as follows:

*I would say that there are three regular forms of communication that [the CSR team] pushes out. There is kind of like an internal social network, which is like a Facebook for employees. They make announcements there. There is also a monthly newsletter, where they highlight what the volunteer opportunities are. And then the third thing is that there are internal articles on our intranet. Then for me, because they know I volunteer a lot, folks from CSR will reach out to me directly and say “Hey, I’m not sure if you’re aware but this is coming up.” So it gets out a variety of ways.* (Business Employee)

Communication touch points up, down, between and *within* business and nonprofit organizations created space to address significant struggles stemming from the clash of cultures that can occur when bringing different sectors together. They also facilitated early the greatest possible clarity between partners about each other's intentions, roles and expectations—even as some of these things may change. Two-way dialogue enabled business leaders, in particular, more visibility into how the network of actors in which the nonprofit is embedded may influence nonprofit decision-making. Finally, regular dialogue allowed partners consistent opportunities to reassess priorities and goals in order to respond with resilience in the inevitable event of change.

(3) Recruit and develop leadership that has experience in both business and nonprofit sectors

Our research indicated that the experiences of people in leadership positions played a key role in making business-nonprofit partnerships work. When partnerships were staffed with people who had experience in both the business and nonprofit sectors, these individuals were able to provide insight for their own organizations about life in their partner's organization. These lived experiences often went beyond basic vocabulary and insights about budgetary or financial constraints. The lived experiences allowed these individuals to work as brokers between the two worlds. In previous work, Aveling and colleagues have referred to “knowledge brokers (52)” as key to partnerships, and Sujin Jang has used the term “cultural brokers” to refer to similarly-situated intermediaries (53, 54). Jang studied more than 2,000 global teams and found that diverse teams with a cultural broker significantly outperformed diverse teams without one. Hence, if we think about inter-organizational partnerships as creating a certain kind of team, the value of cultural brokers is unsurprising. In our cases, their involvement shortened the distance between organizational cultures and increased mutual understanding and responsiveness.

The presence and agency of cultural brokers in both business and nonprofit partner organizations helped sustain strategic partnerships. Our case studies indicated two ways to cultivate cultural brokers within an organization. The first was to hire individuals to work on the partnership who were *themselves* well-networked across sectors because they had experience working in both business and nonprofit settings. The second was to develop closely-knit leadership *teams* composed of people with experience in both sectors. The need for leadership with experience in both domains was not confined to the C-suite but also extends to distributed leadership networks that include individuals who operate at other levels within the company and across diverse organizational units.

No matter the method, businesses that chose to cultivate nonprofit experience within their ranks had useful internal references for their partners’ experiences—as did nonprofit organizations that employed people with business backgrounds. Managers with experience of “other sector” had the ability to speak persuasively to partner concerns and interests, thereby increasing their employers' ability to respond adroitly. One business leader described the value of cross-trained people in helping the business access the best possible information: “*The relationships [with people from other sectors] provide you with information and it's the access and compilation of all of that information that makes you most effective.”*

To optimize leaders' skills and experiences, organizations in our study developed structures and contexts for cultivating their translation capacities. Effective cultural brokers enabled others to tap into their networks, not just by delegating tasks, but supporting others to develop their own relationships within the relevant networks. Cultural brokers relied not just on individual traits (such as charisma and communication skills), but from experiences living, working, and being embedded in diverse networks and sectors. For example, a business senior leader in the Incubation case identified up-and-coming leaders and then encouraged them to attend community events or events with local politicians so that they could begin to build their own networks.

*In my opinion to be truly successful you can't leave out any one of those circles [circles being business, community and philanthropy, and politics], and I think probably the higher up you go inside the organization the more you develop all three deeply, but I would say we still encourage younger people to be familiar and to understand what is going on.*(Business senior leader)

(4) Mobilize non-financial commitments in support of the partnership

In light of the resource disparity that commonly exists between businesses and nonprofit partners, it can be natural for businesses starting strategic partnerships to anticipate the need to make financial investments in their new partners. What we found is that businesses looking to sustain those partnerships will likely also need to invest time, energy and resources in their own intra-organizational capacity. “Cutting checks” was, in some sense, the most straightforward way for a business to support a nonprofit. Doing “more than cutting checks” demanded additional effort on the business' behalf. This included dedicating staff hours, developing information management systems, cultivating the infrastructure to enable employee volunteering, and in some cases, paying for employee time spent at the nonprofit.

In particular, initiatives we observed demanded considerable intra-firm coordination and commitment from across departments. One business took the initiative to create “flash consulting” days, deploying groups of employees to help the nonprofits work through organizational problems identified by the nonprofits. The consulting groups represented diverse segments of business and each group was paired with one nonprofit. They meet for several hours at the end of the dedicated day, made recommendations for the nonprofits' consideration. Another deployed IT staff to help a nonprofit partner develop a new data base. Both activities required considerable planning, oversight and sign-off from various levels of management within the business.

Both public sector and nonprofit partners in our study indicated that the commitment of non-financial resources was an indicator of genuine commitment on the part of business. Nonprofits in our study noted the value of partnering with firms that had well- developed and integrated approaches to CSR (40, 41).

*Int: Think about a strong corporate partner, what do you think contributes to really being able to have a good relationship with them and the ideal level of engagement?*

*Nonprofit Manager: I think [what it is], fundamentally, is having a corporate social responsibility program be kind of embedded in the DNA of the company. It's not checking a box. [..] The organizations or corporations with the most mature CSR program are [the ones] where they've got individuals who are dedicated to this. It's not somebody who just wants to do something good so joins a committee in addition to all of their other job responsibilities*.

On the contrary, we found that nonprofit skepticism about the sincerity of business involvement was often attributable to internal capacity constraints at the business. This is consistent with previous literature that has found that focused engagement allows business' to demonstrate sincerity in their social commitments to customers or regulators (55, 56).

All of the strategies we have described here require dedicated resources. Developing internal capacity for responsive strategic partnering allows organizations to flexibly mobilize internal resources as projects and needs evolve. It also avoids the partnership being siloed in the business’ CSR department or a single nonprofit programmatic area, which can limit the relationship's impact. While cross-sector collaboration is often a difficult and uncertain process, dedicating time and resources is central to building the capacity for responsiveness that collaborators value and that leads to more resilient strategic partnerships.

## Discussion

Studying long-term partnerships between businesses and nonprofits shed new light on what makes them resilient over time. Our four cases encompassed some non-intuitive challenges and novel approaches to managing these cross-sector relationships for the purposes of achieving population health improvements. In contrast to inherited wisdom about business-nonprofit relationships, the insights we gained from our research emphasized the need for business managers to develop a capacity to be responsive to their partners' inherent differences. The four practices that surfaced—tolerating ambiguity and uncertainty, developing robust communication structures, cultivating leaders who could act as cultural brokers and committing non-financial resources towards the work—comprise a new perspective on business-nonprofit partnerships that is routed in organizational learning. Cross-sector partnerships that *learn*, in short, are more resilient in the face of uncertainty than partnerships that *plan*. The latter are destined to encounter surprises, usually unwelcome ones, as a result of their differences in operating modes, which can derail a relationship by violating the expectations captured by the plan. But partnerships that learn expect surprises, learn from them, and continually develop their capacity to work together.

Our findings thus differ from conventional wisdom on what makes partnerships work. We did *not* find evidence in any of our cases of multi-year partnerships of a business and nonprofit trying to match the two organizations' purposes, incentives, or metrics in an effort to align around their shared goal. Celebrating efforts to “align” the organizations (57–59) may seem a logical way to facilitate partnership, but our study suggests that building strategic partnerships on the basis of sameness may be imprudent. It is the *differences* between businesses and nonprofits that make a partnership attractive in the first place. Striving to erase those differences, for the purposes of making the relationship easier to manage, could undermine the value of partnering. This argument mirrors aspects of the strategic alliance literature, which suggest that alliances are most successful when based on synergies rather than similarities (11, 60). In order to leverage the differences between partners, we have suggested that what businesses can do is build internal capacity in order to be responsive to nonprofits.

In highlighting the value of difference, our analysis also drew attention a paradox related to trust in business and nonprofit relationships: when partners are starkly different from one another, they need to rely on trust more heavily to facilitate collaboration but they will find trust more difficult to develop. In other words, trust is critical in these relationships because the partners are unfamiliar with one another, and yet this unfamiliarity makes embarking on a deep, strategic partnership especially risky. Actions required by the partnership—such as sharing sensitive information or making substantial financial investment in an untested idea—leave each partner vulnerable to the other's potential exploitation. The trust inherent in these actions could be violated if a partner chose to distribute that information widely or suddenly back out of the project. Particularly for a business and nonprofit who are unfamiliar with one another—and the wider, influential networks within which each is embedded—it can be unsettling to confront the plausibility of these outcomes.

To make matters more difficult, our data echo previous findings that business and nonprofit partners often come to a potential partnership without a reservoir of trust to draw upon (39, 61). Rather than a neutral stance, partners are likely to have experienced or observed fraught relationships between businesses and nonprofits that create a trust deficit on both sides (62). Past, negative experiences—or even perceptions—could encourage a trust deficit by making partners wary of revealing vulnerabilities, for fear that their partner would exploit them (63). Overcoming such a deficit requires the mistrustful partner to risk exploitation in order to discover a partner can be trusted. But of course, it is natural for management teams to wonder: why should we risk exploitation if we believe our partners to be untrustworthy? This cross-sectoral history highlights the need to develop partnerships incrementally over long time horizons.

We recognize that our suggestions ask managers to withstand, if not embrace, a considerable amount of uncertainty. Although travelling this path of uncertainty and shared learning is challenging for those accustomed to a traditional project management paradigm, it offers an opportunity for mutual learning and the possibility of value creation. It creates particular discomfort in a business domain (CSR) that many still see as supererogatory, which makes it easy for some managers to simply abandon the effort. Yet, uncertainty is increasingly unavoidable—not just in novel partnerships but also in each organization's standard operating environment. Thus, more than just their cross-sector partnerships stand to gain from business and nonprofit mastery of these resilience building strategies.

## Data Availability

The dataset generated and analyzed during the current study are not publicly available because they contain information that could compromise research participant privacy. Anonymized data that support the findings of this study are available on reasonable request from the authors.
